# Selenium as a factor moderating depression and obesity in middle-aged women

**DOI:** 10.1192/j.eurpsy.2025.1368

**Published:** 2025-08-26

**Authors:** D. Schneider-Matyka, A. M. Cybulska, E. Grochans, M. Panczyk, I. Cerzniewska, M. Stanisławska, K. Rachubińska

**Affiliations:** 1 Department of Nursing, Pomeranian Medical University in Szczecin, Szczecin; 2 Department of Education and Research of Health Sciences, Warsaw University, Warszawa, Poland

## Abstract

**Introduction:**

Selenium seems to have the ability to alleviate inflammatory signaling path-ways. Obesity is associated with chronic low-grade inflammation. Depression is also defined as an inflammatory disorder. PPAR-γ has been shown to have antidepressant-like effects The levels of inflammatory cytokines that are parameters associated with obesity―are also related to the severity of depression. Studies confirm an increased risk of depressive symptoms in middle-aged women. Therefore, it seems reasonable to consider the influence of selenium, PPAR-γ, and selected proinflammatory cytokines in the context of obesity and depression among middle-aged women.

**Objectives:**

The aim of this study was to evaluate the effect of serum selenium on PPAR-γ and selected proinflammatory cytokines (IL-1β, IL-6, TNF-α) in relation to depressive symptoms and obesity in middle-aged women.

**Methods:**

The study sample included 443 middle-aged women living in north-western Poland. The research procedure: a survey performed using the authors’ questionnaire and the BDI, anthropometric measurements, and analysis of blood for the levels of selenium, cytokines, and genetic analysis of the PPAR-γ polymorphism.

**Results:**

It has been found that BMI increases along with the concentration of IL-6. No moderating effect of selenium was observed, although cut-off values for “p” were established for IL-β*Se (p=0.068) and IL-6*Se (p=0.068), so there is a potential association with these two markers. At high selenium levels, the effect of higher IL-β levels on a decrease in BMI was stronger. So was the effect of an increase in IL-6 levels on an increase in BMI.

**Conclusions:**

No effect of selenium on PPAR-γ has been found in relation to depressive symptoms and obesity. 2. Higher selenium levels may have a beneficial effect on BMI even at high IL-β concentrations, however, at high IL-6 concentrations, this effect was not observed. 3. Selenium levels had no impact on depressive symptoms.

**Disclosure of Interest:**

None Declared

